# CD19^+^CD1d^hi^CD5^hi^ B Cells Can Downregulate Malaria ITV Protection by IL-10 Secretion

**DOI:** 10.3389/fpubh.2020.00077

**Published:** 2020-03-17

**Authors:** Hongli Guan, Jiacong Peng, Liping Jiang, Gang Mo, Xiang Li, Xiaohong Peng

**Affiliations:** Department of Parasitology, Guilin Medical University, Guilin, China

**Keywords:** Plasmodium, memory B cells, B10 cells, infection treatment vaccine (ITV), IL-10, malaria vaccine

## Abstract

Infection treatment vaccine (ITV) can lead to sterile protection against malaria infection in mice and humans. However, parasite breakthrough is frequently observed post-challenge. The mechanism of rapid decline in protection after the last immunization is unclear. Herein, C57BL/6 mice were immunized with 10^3^, 10^5^, or 10^7^ ITV thice at 14-day intervals. Mice were challenged with 10^3^ parasites at 1, 3, and 6 months after last immunization and the protection was checked using blood smear. The phenotypes of B cells were analyzed by flow cytometry. The levels of serum cytokines were quantified using cytometric bead array. The 10^3^ ITV vaccination group exhibited 100% protection at 1 month after last immunization, and the 10^5^ group showed sterile protection at 3 months after last immunization. However, the 10^7^ group showed only partial protection. Further, the protection declined to 16.7% at 6 months after last immunization in 10^5^ and 10^7^ groups, whereas it maintained for more than 60% in 10^3^ group. The number of memory B cells (MBC) decreased along with the decline in protection. However, programmed cell death protein 1 (PD-1) expressed on MBCs did not show significant variation among the three groups. Interestingly, CD19^+^CD1d^hi^CD5^hi^ B cells, defined as B10 cells, exhibited negative regulation with respect to protection. The numbers of CD19^+^CD1d^hi^CD5^hi^ B cells in the 10^3^ group at 1 months and in the 10^5^ group at 3 months post-immunization were the lowest compared to those in the other groups. Moreover, the serum levels of interleukin 10 (IL-10) in these two groups were also significantly lower than those in other groups. We conclude that higher immunization dose may not lead to better protection with the malaria vaccine as CD19^+^CD1d^hi^CD5^hi^ B cells can downregulate ITV protection against malaria via IL-10 secretion. These results could facilitate the design of an effective long-lasting malaria vaccine with the aim of maintaining MBC function.

## Introduction

Malaria is still one of the three most important infectious diseases worldwide, resulting in 228 million clinical cases and 405,000 deaths in 2018, mostly in Africa in children under 5 years of age (1). The emergence and spread of insecticides and anti-malarial drug resistance (2) have posed severe challenges in the prevention and control of malaria. An effective and long-lasting malaria vaccine is urgently needed to eliminate this disease.

The blood stage of the malarial parasite life cycle is responsible for all the clinical symptoms of malaria (3). The goal of blood stage malaria vaccines is to inhibit the proliferation of intraerythrocytic malaria parasites, so as to control the symptoms of malaria and prevent the disease. The protection of blood stage malaria vaccines depends on acquisition of antibodies against parasite target antigens (4). However, maintaining the antibodies is still a bottleneck for effective vaccine design. Infrequent malaria infections can induce antigen-specific, long-lived antibody, and antigen-specific memory B cell (MBC) responses in a significant proportion of malaria-exposed individuals (5). The role of MBCs in the maintenance of protection conferred by the vaccine has also been confirmed in a mouse model (6). However, the protection conferred by blood stage vaccines is not sterile and quickly wanes if an individual leaves the endemic area (7). Therefore, inadequate maintenance of the function of long-term, effective malaria parasite-specific MBCs is an urgent problem that needs to be solved.

A malaria infection-treatment-vaccine (ITV) as anti-malaria drug prophylaxis is effective against the blood stage of Plasmodium (8). Human experiments have confirmed that ITV can induce sterile protection against homogeneous *Plasmodium falciparum-*infected mosquitoes, and this protection can last for approximately 2 years (9, 10). Related studies have shown that ITV immunization with an extremely low dose of the parasite (in cases where no parasitemia has been found in routine blood smears) can induce stronger protection than drug control after parasite infection (11). This indicates that low-dose immunization may confer better protection.

Over the past decade, a number of studies have demonstrated that regulatory B cells (Bregs) are crucial in the maintenance of immune tolerance and suppression of inflammation (12). B10 cells, a Breg subset, have been shown to limit immune response to pathogen infection via the release of interleukin-10 (IL-10) (13). CD19^+^CD1d^hi^CD5^hi^ B cells are defined as B10 cells in mice (14–16) as well as CD19^+^CD24^hi^CD38^hi^ B cells in humans (17). The number of B10 cells is significantly increased during acute infections resulting in decrease in inflammation (18). However, B10 cells are functionally impaired or their abundance is lower in autoimmune diseases or chronic infections (19–21). Thus, we speculate that high-dose *Plasmodium* immunization induces B10 cells to increase in number similar to that in acute infections, and subsequently accelerates the decline in protection.

In this study, we mainly explored the role of MBCs and B10 cells in immune dose-mediated long-term protection decline of malaria blood stage ITV, and clarified the role of PD-1 in MBC and B10-related cytokines in this process. This study will thus provide a new research direction to explore the mechanism of decline in long-term protection of ITV, and provide a theoretical basis for improving the long-term protection of malaria vaccine as well as for the design and application of a more effective vaccine.

## Methods

### Mice and Malaria Parasite Strain

Female C57BL/6 mice and BALB/c mice were obtained from the Hunan Silaike Jingda Laboratory Animal Co. Ltd. All mice ranged in age from 6 to 8 weeks when the experiments were initiated. All mice were maintained in the experimental animal centers of Guilin Medical University. The lethal strain of *Plasmodium yoelii* 265 was originally obtained from the Department of Human Parasitology at Guilin Medical University and was maintained as cryopreserved stabilates. All animal studies were reviewed and approved by the Animal Ethics Committee of the Guilin Medical University Institute of Medical Research.

### Vaccination

First, cryopreserved *P. yoelii 265* were thawed and 100 μL of this suspension was inoculated into mice by intraperitoneal (i.p.) injection. Four days later, the blood of infected mice was harvested by cardiac puncture and parasitemia was determined. Then, naïve mice were immunized thrice by intravenous (i.v.) injection with a 10^3^, 10^5^, or 10^7^ dose of *P. yoelii* 265-infected red blood cells (Py-iRBCs) at 2-week intervals. All mice were then i.p. injected with 100 μL of 8 mg/mL chloroquine (CQ; Sigma-Aldrich, St. Louis, MO, USA) diluted in saline daily for 14 days, starting on the day of iRBC injection. The absence of parasites was confirmed by Giemsa-staining of blood smears from all treated mice since the beginning of CQ treatment.

### Challenge

Before challenge, the absence of blood stage parasite infection was confirmed by Giemsa staining of thin blood smears. Mice were challenged with 10^3^ Py-iRBCs by intravenous (i.v.) injection at 1, 3, and 6 months (mo) after the last immunization. Blood stage infection was examined daily from 3 days post-challenge to the days until parasitemia disappeared or the mice died. Parasitemia was calculated as the percentage of iRBCs.

### Flow Cytometric Analysis

Spleens were collected at 1, 3, and 6 months after the last immunization, and splenocytes were prepared as described previously (8). Phenotypic analysis of lymphocytes was performed by flow cytometry. Cells were stained using fluorescein isothiocyanate (FITC)-conjugated anti-mouse CD19, PE-Cy5.5 anti-mouse-CD27, and phycoerythrin (PE)-conjugated anti-mouse-CD279 [also known as programmed cell death protein 1 (PD-1)] to detect the PD-1 expression on MBCs (**Figure 2A**). B10 cells were stained with FITC-conjugated anti-mouse CD19, PE-conjugated anti-mouse-CD5, and allophycocyanin (APC)-conjugated anti-mouse-CD1d. All antibodies were purchased from BioLegend.

Initially, 10^6^ cells were resuspended in 50 μL fluorescence-activated cell sorting (FACS) buffer (phosphate-buffered saline (PBS) supplemented with 2% heat-inactivated fetal bovine serum (FBS); Gemini Bio-Products). Then, the mixture was incubated for 10–15 min at 4°C. Next, 50 μL of the 2X antibody cocktail was added to each tube (for the unstained sample, 50 μL of FACS buffer was added), vortexed gently or tapped to mix, and incubated at 4°C for 20–40 min in dark. Next, after washing with FACS buffer, 200 μL FACS buffer was added to resuspend the cells. The cells were analyzed using FACSCanto II instrument (BD Biosciences, San Jose, CA, USA), and the data were analyzed with FlowJo version 10 software.

### Serum Cytokine Detection

The whole blood of mice was obtained by cardiopuncture before euthanization, and serum was harvested by centrifugation. The levels of the proinflammatory cytokines interleukin 6 (IL-6), monocyte chemoattractant protein-1 (MCP-1), interferon γ (IFN-γ), tumor necrosis factor α (TNF-α), IL-12p70, and the anti-inflammatory cytokine, IL-10, in serum samples were quantified using the cytometric bead array (CBA) Mouse Inflammation Kit (BD Biosciences), according to the manufacturer's instructions. Briefly, mouse inflammation standards were prepared with 2 mL of assay diluent, and by doubling the dilution to 1:2, 1:4, 1:8, 1:16, 1:32, 1:64, 1:128, 1:256, 1:512, and 1:1,024. Six mouse inflammation capture beads were mixed thoroughly. Next, 50 μL of mixed capture beads were incubated with the same volume of each mouse inflammation standard dilution or each sample. Then, 50 μL mouse inflammation PE detection reagent was added to all assay tubes and incubated for 2 h at room temperature, in dark. After washing with washing buffer, samples were analyzed on a FACSCanto II instrument (BD Biosciences), and the data were analyzed with FCAP Array version 3 software (22).

### Statistical Analysis

The data were analyzed using GraphPad Prism version 5 software. Non-parametric tests (Mann-Whitney test) and two-way analysis of variance (ANOVA) were used to compare groups, and *P* < 0.05 were considered statistically significant. Pearson correlation analysis was performed using SPSS 17.0.

## Results

### C57BL/6 Mice Showed Quicker Protection Decline Than BALB/c Mice

First, we investigated the protection against *P. yoelii* challenge in different mice strains. C57BL/6 and BALB/c mice were immunized with 10^5^ *P. yoelii* ITV and challenged with 10^3^ homogeneous iRBC at 1, 3, and 6 months after the last immunization. As shown in Table 1, both C57BL/6 and BALB/c mice showed sterile protection against 10^3^ iRBC challenge at 1 month. However, the protection quickly dropped to 40% in C57BL/6 mice at 3 months, whereas it was maintained in BALB/c mice. At 6 months, C57BL/6 mice only showed 20% protection compared to 80% in BALB/c mice.

**Table 1:** Protection against 10^3^ *Plasmodium yoelii* 265-infected red blood cells (iRBC) challenge in infection treatment vaccine (ITV)-immunized mice of two strains—BALB/c and C57BL/6.

**Mice**	**Immunogens**	**1 month**	**3 months**	**6 months**
BALB/c	PBS	0% (0/5)	0% (0/5)	0% (0/5)
	10^5^ ITV	100% (5/5)	100% (5/5)	80% (4/5)
C57BL/6	PBS	0% (0/5)	0% (0/5)	0% (0/5)
	10^5^ ITV	100% (5/5)	40% (2/5)	20% (1/5)

### Low Immunization Dose Group Gained Better Protection Than High-Dose Group

**Figure 1:**
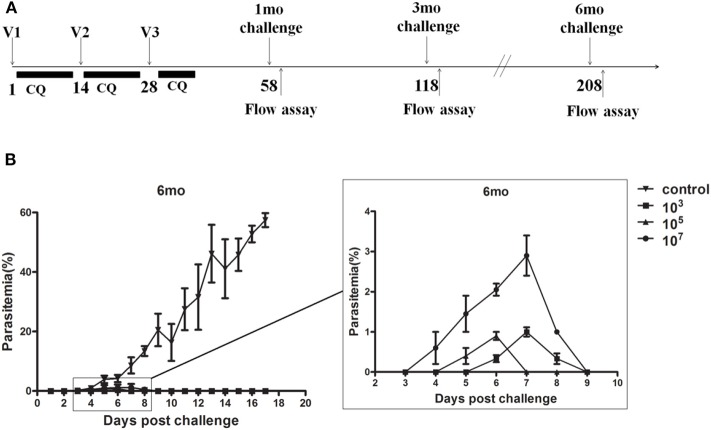
**(A)** The procedure for ITV immunization and experiment operation. **(B)** Naive or immunized mice (*n* = 5) were challenged intravenously (i.v.) with 10^3^ *Plasmodium yoelii* 265-infected red blood cells (iRBC) at 6 months. Parasitemia was recorded. The results are representative of two independent experiments. The Y-axis shows the percentage of erythrocytes infected with parasites, as determined by microscopy of Giemsa-stained thin blood smears, and the X-axis corresponds to days post-infection (p.i.). The data are presented as mean ± standard deviation (SD).

To explore the protection resulting from immune doses, we immunized groups of C57BL/6 mice with 10^3^, 10^5^, and 10^7^ ITV and challenged them with 10^3^ parasite iRBCs by i.v. injection at 1, 3, and 6 months after the last immunization (Figure 1A). Parasitemia was detected by thin blood film smear from 3 days post-challenge until the parasitemia disappeared or the mice died, and parasitemia was calculated as the percentage of iRBCs. The protection rates and peak parasitemia of the three immunized groups at three different times were compared after a blood stage challenge, as depicted in Table 2.

**Table 2:** Protection assay of different immunization dose groups.

**Groups**	% Protection (peak parasitemia^*^)		
	**1 month (*n* = 10)**	**3 months (*n* = 10)**	**6 months (*n* = 6)**
Control	0% (>60%)	0% (>60%)	0% (>60%)
10^3^	100% (–)	60% (0.50%)	66.7% (1.00%)
10^5^	60% (0.5%)	100% (–)	16.7% (0.90%)
10^7^	20% (1.86%)	70% (2.50%)	16.7% (2.90%)

* *C57BL/6 mice were immunized three times with 10^3^, 10^5^, or 10^7^ ITV at 2-week intervals and were challenged with 10^3^ parasite iRBCs by i.v. injection at 1, 3, and 6 months after last immunization. Peak parasitemia represents the highest parasitemia of challenged mice. Data of two independent experiments combined are exhibited*.

Mice in the control group showed detectable blood stage parasitemia 4 days after challenge and died at 19–21 days post-challenge. However, in immunized groups, the infection started from 3 to 7 days post-challenge and lasted with low-dose parasitemia only for 3–6 days (Figure 1B). The peak parasitemia gradually increased over time in all three groups. Remarkably, the 10^7^ group revealed the highest peak parasitemia compared to the other two lower dose groups. In addition, the 10^3^ group gained sterile protection in the 1-month challenge, and maintained more than 60% protection for at least 6 months. However, the 10^5^ and 10^7^ immunized groups only exhibited 16.7% protection after 6 months (Table 2).

In other words, malaria blood stage ITV long-term protection of the 10^7^ group was generally worse than that of the 10^3^ and 10^5^ groups, and the long-term protection of ITV decreased with the prolongation of immunization time.

### CD19^+^CD27^mid^ Memory B Cells Decreased With the Prolongation of Immunization Time

**Figure 2:**
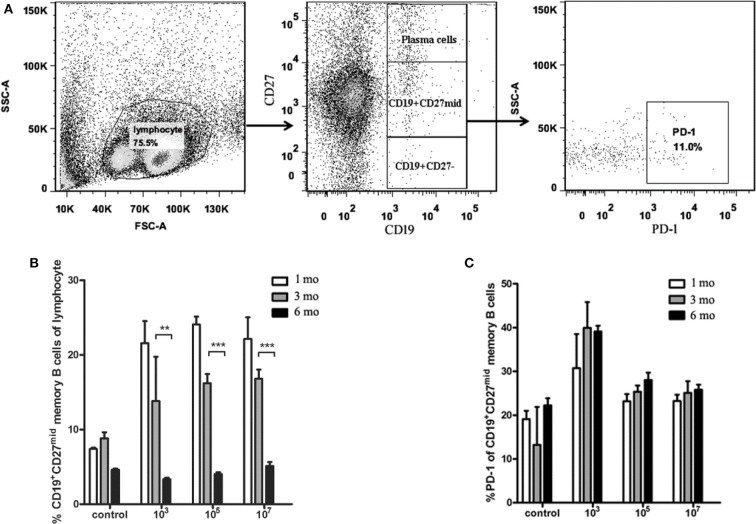
The number of memory B cells and PD-1^+^ memory B cells in the spleens of the ITV-immunized mice. **(A)** Gating strategy of PD-1^+^ memory B cells. **(B)** The number of memory B cells declined with immunization time in each group. **(C)** PD-1^+^ memory B cells exhibited no significant variation at different observation time points. Two individual experiments were performed and combined. The data are presented as mean ± SD. **p < 0.01 and ***p < 0.005.

To characterize the protection resulting from immunization dose, we first observed the number of MBC variations in spleen. The results showed that the number of CD19^+^CD27^mid^ MBCs in the three experimental groups decreased gradually over time (Figure 2B). The number of MBCs in all three groups at 6 months was significantly lower than that at 3 months (10^3^: *p* = 0.0023; 10^5^: *p* = 0.0007; 10^7^: *p* = 0.001), but the number of MBCs at 3 months was not significantly different from that at 1 month (*p* > 0.05). Next, we analyzed if PD-1 expressed on MBCs played a role in the difference in protection. We found no significant difference in PD-1 expression on CD19^+^CD27^mid^ MBCs in the three immunized groups at different times (*p* > 0.05; Figure 2C). This result indicated that the number of MBCs decreased with the prolongation of immunization time.

### Low Immunization Dose Group Displayed Low Number of CD19^+^CD1d^hi^CD5^hi^ B Cells During Sterile Protection Phase Compared With the High Immunization Dose Group

**Figure 3:**
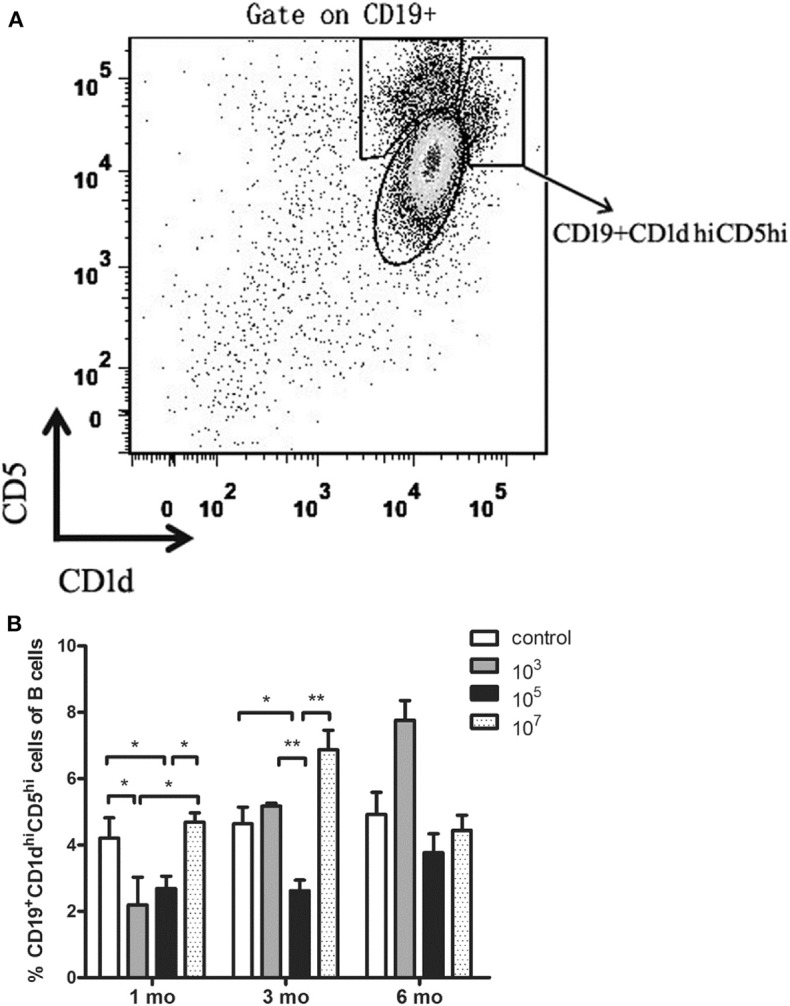
**(A)** Gating strategy of CD19^+^CD1d^hi^CD5^hi^ B cells. **(B)** Statistical analysis of the total number of CD19^+^CD1d^hi^CD5^hi^ B cells in the spleen from ITV-immunized mice at 1, 3, and 6 months after the last immunization. Data from two independent experiments combined is exhibited. The data are presented as mean ± SD. *p < 0.05 and **p < 0.01.

In order to explore whether the immune dose could affect the increase in B10 cell abundance, splenocytes were collected and the number of CD19^+^CD1d^hi^CD5^hi^ B cells was counted by flow cytometry. The results showed that at 1 month, the number of B10 cells of ITV-immunized mice in 10^3^ and 10^5^ groups was significantly lower than that in the control group (10^3^: *p* = 0.0497, 10^5^: *p* = 0.0232), and that in the 10^7^ group was significantly higher than that in the 10^3^ (*p* = 0.0471), and 10^5^ (*p* = 0.0128) groups (Figure 3). This was in accordance with the protection assay at 1 month. At 3 months, the number of B10 cells in 10^5^ group was significantly lower than that in control (*p* = 0.0275), 10^3^ (*p* = 0.0075), and 10^7^ groups (*p* = 0.0071).

The results showed that the immunized group with the best protective effect expressed the lowest number of B10 cells. This suggests that the expansion of B10 cells in ITV-immunized mice may result in reduced long-term protective efficacy of ITV.

### The Amount of IL-10 in Mouse Serum Was Positively Correlated With the Number of B10 Cells, Whereas the Amounts of IL-6 and IFN-γ Were Negatively Correlated With the Amount of IL-10

**Figure 4:**
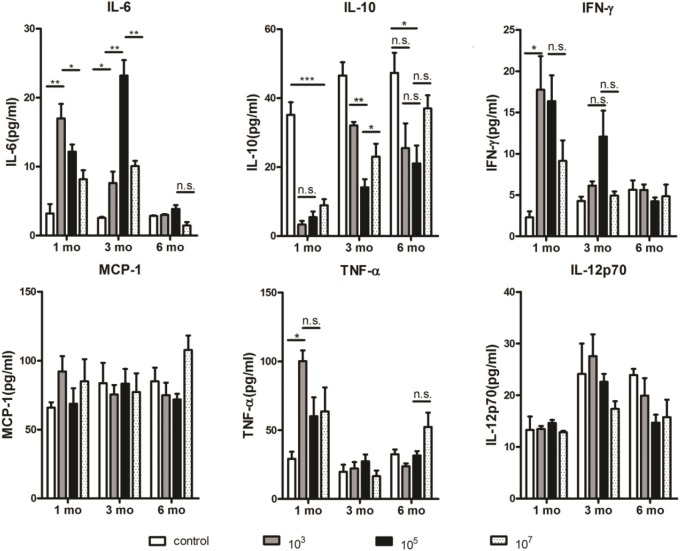
At 1 and 3 months, the amount of IL-10 in the 10^3^ and 10^5^ groups was the lowest, respectively. Serum samples were collected from immunized mice at the indicated time points after the final immunization and the concentrations of cytokines were measured serially by a cytometric bead array (CBA). Two individual experiments were performed. The data are presented as the mean ± SD. Data were compared with the two-way analysis of variance (ANOVA). *p < 0.05, **p < 0.01, ***p < 0.005, and n.s., not significant.

Considering the important role of cytokines in the immunosuppressive effects of B10 cells (23–25), cytokine levels in serum were investigated. The concentrations of IL-6, MCP-1, IFN-γ, TNF-α, IL-12p70, and IL-10 in serum were measured by CBA. As shown in Figure 4, the level of IL-10 in the control group was significantly higher than that in the three immunized groups, especially at 1 month (10^7^: *p* = 0.0029). The level of IL-10 in the 10^5^ group was lower than that in the 10^3^ (*p* = 0.0155) and 10^7^ groups (*p* = 0.0022) at 3 months. However, no significant variation was found among the three experimental groups at 6 months. Therefore, Pearson correlation analysis showed that the level of IL-10 was proportional to the number of B10 cells (*r* = 0.41, *p* = 0.015). Interestingly, the amount of IL-6 was inversely proportional to the amount of IL- 10 (*r* = −0.626, *p* < 0.001). At 1 and 3 months, the amount of IL- 6 in the control group was significantly lower than that in the three experimental groups. The level of IL-6 in the 10^3^ group was higher than that in the control (*p* = 0.0053) and 10^7^ (*p* = 0.0236) groups at 1 month. Meanwhile, the 10^5^ group exhibited higher IL-6 levels than the 10^3^ (*p* = 0.0051) and 10^7^ (*p* = 0.0052) groups at 3 months. No significant difference was found at 6 months among all the four groups.

In addition, the level of IFN-γ was also inversely proportional to the level of IL-10 (*r* = −0.638, *p* < 0.001). The level of IFN-γ in the control group was significantly lower than that in 10^3^ group (*p* = 0.011) as well as other immunized groups at 1 month. Although no significant difference was found among three immunized groups at different time points, 10^5^ group tended to express higher IFN-γ level than other three groups at 3 months. The levels of TNF-α, MCP-1, and IL-12p70, which could be negatively regulated by IL-10 (26–28), were not significantly correlated with the level of IL-10.

Taken together, the amount of IL-10 in mouse serum was positively correlated with the number of B10 cells, whereas the amount of IL-6 and IFN-γ was negatively correlated with the amount of IL-10. Thus, these data suggest that increased level of IL-10 may lead to decline in protection. The role of IL-6 and IFN-γ in this process needs to be verified by further experiments.

## Discussion

All clinical symptoms of malaria mainly occur due to the blood phase of the malaria parasite life cycle (3). Therefore, the goal of blood stage malaria vaccines is to inhibit the proliferation of intraerythrocytic malaria parasites so as to control the symptoms of malaria and prevent the disease. However, there is still no effective malaria vaccine for humans because the protection of blood stage vaccine is not sterile and wanes quickly; moreover, there may be a risk of malaria parasite infection caused by the vaccine (29). Thus, exploring the mechanism of long-term protection decline of malaria vaccine will be helpful for designing a more effective malaria vaccine. Our previous results have shown that the long-term protective efficacy of malaria vaccine in the low immunization dose group was better than that in the high immunization dose group (30). Previous studies have shown that ITV immunized at an extremely low dose of the parasite can induce stronger protection than high doses (11). Therefore, it was unclear whether the immunization dose also affects the long-term protection of the blood stage malaria vaccine.

Herein, we explored the influence of immune dose on Plasmodium ITV protection longevity. C57BL/6 mice are more sensitive to *P. yoelii* than other mouse strains. Theoretically, they could generate more antigens than other mouse stains. However, our experiment found that ITV-immunized C57BL/6 mice have a quicker protective decline than BALB/c mice (Table 1). Thus, C57BL/6 mice were chosen for further dosage associate investigation. C57BL/6 mice were immunized with different doses of parasites and were challenged at 1, 3, or 6 months post-immunization. Although immunization with different dosages conferred partial protection against the challenge, variations in protection were observed. The highest immunization dose, the 10^7^ group, showed the highest peak parasitemia and lowest protection rate at all three challenge phases (Table 1, Figure 1B). However, it is challenging to compare the 10^3^ and 10^5^ groups because the 10^3^ group showed sterile protection at 1 month, whereas the 10^5^ group exhibited 100% protection at 3 months. Nevertheless, the 10^5^ group had a lower protection rate than the 10^3^ group at 6 months. These results indicated that the immunization dose could mediate the long-term protection of malaria ITV in C57BL/6 mice, and that a higher immunization dose could not exert a better long-term protective effect.

Studies have found that splenic MBCs played the most important role in the immune protection induced by Plasmodium blood stage ITV vaccine. The number of malaria parasite-specific MBCs is proportional to the immune protection of the host, and decrease of the number of specific MBCs leads to decrease in immune protection (5). Our results also showed that with the increase in immunization time, the number of MBCs decreased gradually in all the three immunization groups. Therefore, the regression of ITV long-term protection may be related to the decrease in the number of MBCs of mice with an increase in immunization time.

B10 cells, a small subset of CD19^+^CD24^hi^CD38^hi^ B cells as well as CD19^+^CD1d^hi^CD5^hi^ B cells in mice, are an immunosuppressive B cell type that stop the expansion of pathogenic, proinflammatory lymphocytes and play a significant role in suppressing autoimmune responses and preventing autoimmunity through the secretion of IL-10, IL-35, and transforming growth factor β (TGF-β) (31). Previous studies have shown that the number of B10 cells is significantly increased during acute infection, resulting in decreased inflammation (18). However, B10 cells are functionally impaired or their abundance is decreased in autoimmune disease or chronic infection (19–21). Therefore, we hypothesize that high-dose *Plasmodium* immunization could induce B10 cells to increase in number and subsequently mediate a decline in protection. Our results showed that the number of B10 cells in the 10^3^ and 10^5^ group mice was the lowest at 1 and 3 months, respectively. This was in accordance with the protection results. The number of B10 cells in the 10^7^ group was higher than that in the 10^5^ and 10^3^ groups at 1 and 3 months, respectively, after the last immunization. Meanwhile, the number of B10 cells in the 10^3^ and 10^5^ groups at 6 months was higher than that at 1 and 3 months. This confirmed that B10 cells could downregulate the long-term protection conferred by ITV. Therefore, it can be concluded that worse ITV long-term protection mediated by high immunization dose is related to an increase in the number of B10 cells in the mouse spleen.

A previous study indicated that the main mechanism of the inhibitory action of B10 cells is IL-10 production (32). In addition, some studies indicate that increased IL-10 levels are accompanied by decreased levels of other cytokines, such as IL-6 and IFN-γ (33, 34). Our results thus show that the amount of IL-10 in the 10^3^ and 10^5^ groups of mice was the lowest at 1 and 3 months, respectively. This correlated with the number of B10 cells. On the contrary, the amount of IL-6 and IFN-γ of the 10^3^ and 10^5^ groups was the highest at 1 and 3 months, respectively. This was negatively correlated with the amount of IL-10. Therefore, an increased level of IL-10 secreted by B10 cells may lead to a decline of long-term protection by malaria ITV. However, the role of IL-6 and IFN- γ in this process needs to be verified by further experiments.

In conclusion, we demonstrated that the immunization dose could mediate the long-term protection of Plasmodium blood stage ITV in C57BL/6 mice, and that a higher immunization dose could not produce a better long-term protective effect. The long-term protection of ITV was shown to be related to the increase in the number of CD19^+^CD1d^hi^CD5^hi^ B (B10) cells, which may negatively regulate the long-term protective effect of malaria ITV by secreting IL-10. In addition, the decrease in long-term protection of ITV may be related to the decline of MBCs in the spleen of mice with an increase in immunization time. Our study thus elucidates the mechanism of high immunization doses inhibiting long-term immunity protection in a model of long-term protection decline of malaria blood stage ITV mediated by the immunization dose due to the regulatory role of B10 cells, thus providing a theoretical basis for the design of malaria vaccine.

## Data Availability Statement

All datasets generated for this study are included in the article/supplementary material.

## Ethics Statement

This animal study was reviewed and approved by Animal Ethics Committee of the Guilin Medical University Institute of Medical Research.

## Author Contributions

HG, JP, and XL performed this study. HG analyzed the data. LJ collected samples. GM provided technical support. XP designed this work, supervised this study, and final revised the manuscript. HG drafted the work. JP, XL, LJ, and GM revised the work. All of the authors listed have approved the final version for published and agreed to be accountable for all aspects of the work in ensuring that questions related to the accuracy or integrity of any part of the work are appropriately investigated and resolved.

### Conflict of Interest

The authors declare that the research was conducted in the absence of any commercial or financial relationships that could be construed as a potential conflict of interest.
